# Association between pH regulation of the tumor microenvironment and immunological state

**DOI:** 10.3389/fonc.2023.1175563

**Published:** 2023-07-10

**Authors:** Masahiro Hosonuma, Kiyoshi Yoshimura

**Affiliations:** ^1^ Department of Clinical Immuno Oncology, Clinical Research Institute for Clinical Pharmacology and Therapeutics, Showa University, Tokyo, Japan; ^2^ Department of Pharmacology, Showa University School of Medicine, Tokyo, Japan; ^3^ Pharmacological Research Center, Showa University, Tokyo, Japan; ^4^ Division of Medical Oncology, Department of Medicine, Showa University School of Medicine, Tokyo, Japan

**Keywords:** pH, Warburg effect, lactate, immune escape, tumor microenvironment

## Abstract

The tumor microenvironment (TME) is characterized by interactions among various cells, including tumor cells, immune cells, stromal cells, and blood vessels mediated by factors such as cytokines and metabolites. The development of cancer immunotherapy in recent years has facilitated a more comprehensive understanding of the TME. The TME changes with cancer type and host immune status, as well as with therapeutic intervention. However, studies on pH regulation of the TME have been mostly based on lactate, a metabolite of tumor cells. Notably, the Warburg effect results in the increased production of secreted lactate, thereby acidifying the extracellular microenvironment and affecting the surrounding cells. Lactate inhibits the activation and proliferation of CD8+ T cells, M1 macrophages, natural killer (NK) cells, and dendritic cells, contributing to tumor cell immune escape. It is also involved in angiogenesis and tissue remodeling, as well as promotes tumor growth and invasion. In this review, we have discussed the lactate-based pH regulation in tumor cells in the TME and its effects on the other constituent cells.

## Adjustment of acid-base balance

In humans, the extracellular fluid has a slightly alkaline pH of 7.40 ± 0.02. This corresponds to an H+ ion concentration of 40 ± 2 nEq/l and is regulated by the excretion of volatile and non-volatile acids produced in the body. Volatile acids, such as H_2_CO_3_, are produced as CO_2_ from carbohydrates and fats, and approximately 15,000 mEq/day is discharged from the lungs. Conversely, non-volatile acids include approximately 100 mEq/day of amino acid metabolites and 30 mEq/day of phosphoric acid, a metabolite of nucleic acids and ATP. Approximately 70 mEq/day (approximately 1 mEq/kg body weight) of non-volatile acids are neutralized by the kidneys using approximately 60 mEq/day of bases derived from the diet. The non-volatile acids produced are promptly scavenged by buffering substances, thereby minimizing pH alterations. The bicarbonate buffering system accounts for 60% of the extracellular fluid, the bone buffering system, and Hb buffering system outside the cells, whereas the ${\text{HPO}}_{4}^{2-}$ and protein buffering systems are responsible for buffering inside the cells. However, in pathological conditions such as ischemia, inflammation, and systemic respiratory failure, these buffer systems are dysregulated, thereby leading to local and occasionally systemic acidemia (1). The gastrointestinal tract controls the physiological pH by regulating the local neutral range. Appropriate pH adjustment for local enzyme activation regulates digestive function, especially because the different parts of the digestive tract have different pH ranges (stomach pH 1.5–2, duodenal pH 3–5, small intestinal pH 6, and large intestinal pH 7) (2). Furthermore, an appropriate pH promotes diversity in the gut microbiota, which produces metabolites that skillfully regulate host immunity (3).

Osteoclasts promote an acidic microenvironment during bone resorption. Osteoclasts adhere to the bone at the sealing zone, which consists of polymerized actin; the demineralization of the bone is promoted by releasing acid from cells through proton pumps (4). These instances demonstrate the mechanisms by which living organisms maintain their functions by adequately adjusting local acid-base balance under physiological conditions.

## Acidification mechanisms in the tumor microenvironment

The extracellular pH, which is maintained at pH 7.4 in normal tissues, decreases to approximately pH 6.8 in tumors. Decreased extracellular pH in tumors has been reported in epithelial tumors such as lung cancer, breast cancer, and melanoma, as well as in non-epithelial tumors such as sarcoma (5–10). However, tumor tissue is not uniformly acidic, and the pH varies from near neutral to strongly acidic (11–14). This acidic TME is primarily attributed to hypoxia and increased lactate levels owing to increased glycolysis in cancer cells. The TME has a lactate concentration of 10–30 mM, whereas that under physiological conditions is approximately 1.5–3.0 mM (15).

Hypoxia is presumed to be caused by tumor vascular abnormalities. Endothelial cell adhesion is looser in tumor blood vessels than in normal blood vessels, resulting in increased vascular permeability. Furthermore, the thickness of type IV collagen, which constitutes the vascular basement membrane, is varied and irregular in tumors depending on the site of the blood vessel; therefore, tumor blood vessels have varying diameters and random vasculature; thus, tumor arteries, veins, and capillaries lack a hierarchical structure compared with that of normal blood vessels (16). Consequently, cancer tissues have low blood flow despite the abundance of blood vessels, thereby creating a hypoxic microenvironment within the tumor tissue. Increasing dysfunctional tumor blood vessels does not improve the hypoxic microenvironment but promotes tumor growth (17). The interaction between blood vessels and cells within the tumor further promotes angiogenesis and tumor growth, thereby inducing a hypoxic environment (18, 19).

In the hypoxic environment, increased stability of hypoxia-inducible factor 1 (HIF1) results in increased glycolysis and a subsequent decrease in extracellular pH (20). HIF1 stabilization promotes glucose uptake and metabolism by enhancing the expression of glucose transporter type 1 (GLUT1). This metabolic process produces ATP, which increases the levels of lactate and protons (H+), consequently resulting in decreased intracellular pH. To maintain a constant intracellular pH, membrane proteins such as the Na+/H+ exchanger isoform 1 (NHE1) and ATPase and monocarboxylate transporters 1, 4 (MCT1, 4) excrete lactate and protons outside the cell, resulting in a decrease in external pH (21, 22). Notably, the TME lactate is presumed to increase tumor angiogenesis by promoting CXCL8 production from vascular endothelial cells, thereby exacerbating hypoxic conditions and reducing the extracellular pH (23).

## Adaptation of tumor cells to the acidic tumor microenvironment

In 1924, Otto H. Warburg proposed a phenomenon known as the Warburg effect, in which cancer cells exhibit increased lactate production in an aerobic environment; this opposes the Pasteur effect, which reports the suppression of lactate production by oxygen. However, the Warburg effect does not indicate suppression of aerobic respiration, and mitochondrial aerobic respiration in cancer is enhanced compared with that in normal tissue (24). Furthermore, intracellular acidification inhibits enzymes, such as phosphofructokinase-1, involved in glycolysis; however, decreasing the TME pH does not necessarily promote glycolytic metabolism. Nonetheless, oxidative phosphorylation results in the production of 36 ATP molecules per glucose molecule, whereas glycolysis results in the inefficient production of 2 ATP molecules. Therefore, the preference for inefficient glycolytic metabolism in cancer cells has been actively investigated. The Warburg effect is a bona fide phenomenon observed *in vitro* and *in vivo* in animal models and patients with cancer (25). Furthermore, H+ accumulation occurs in the non-hypoxic regions of the tumors, suggesting that cancer cells purposefully select aerobic glycolysis depending on the time and environment (11, 12, 26). Aerobic glycolysis utilizes glycolytic intermediates for the *de novo* synthesis of nucleotides, lipids, and amino acids required for cell proliferation and, together with TCA cycle metabolites, supports tumor growth (24, 27–29). Thus, tumor cells increase lactate production and induce a decrease in extracellular pH, whereas intracellular pH remains unaltered or is slightly higher than that of normal cells.

Tumor cell pH is determined by anion exchangers (SLC4A1, SLC4A2, and SLC4A3), proton transporter vacuolar ATPase (V-ATPase), mono-carboxylate transporters (MCT1, MCT2, MCT3, and MCT4), chloride/bicarbonate exchanger (SLC4A8), and the Na+/H+ exchanger 1 (SLC9A1), NHE1, Na+/K+ ATPase pump, H+/Cl− symporter, and carbonic anhydrase (CA) (22, 30). Furthermore, the transitional utilization of lactic acid has been reported. Metastatic breast cancer cells found in bone produce lactate, suggesting that they promote osteoclast differentiation and metastatic niche formation (31). Furthermore, in glioma cells, lactate stimulates transforming growth factor-β2 (TGF-β) expression, a key regulator of cancer cell migration, invasion, epithelial-to-mesenchymal transition, and metastatic niche formation (32). Furthermore, as elucidated later in the text, an acidic environment inhibits the action of anti-tumor immune cells, including T lymphocytes, natural killer cells, and M1 macrophages. Conversely, it activates immunosuppressive cells such as regulatory T cells and M2 macrophages. Glycolytic selection in the aerobic environment of tumor cells is not necessarily favorable for cancer cell growth *per se.* By creating an acidic environment, acid-induced immunosuppression is relatively beneficial and may form a favorable tumor microenvironment for cancer.

## Adaptation of T and NK cells to the acidic tumor microenvironment

Effector T cells (CTL), which play a crucial role in anti-tumor immune responses, differentiate and proliferate from naive CD8+ T cells *via* stimulation from IL-2 produced by CD4+ T cells presented with cancer antigens by dendritic cells. Activated CTLs kill cancer cells by producing IFN-γ and perforin. In contrast, regulatory T cells (Treg), which are immunosuppressive, play a critical role in immune tolerance and avoid immune responses against self while suppressing anti-tumor immune responses by CTLs. In humans, Tregs are mainly released from the thymus to the periphery as naïve Tregs and transform into effector Tregs upon antigen stimulation. Effector Tregs suppress the maturation of antigen-presenting cells, consume IL-2, and produce inhibitory cytokines (such as TGF-β and IL-10), thereby suppressing the activation of cytotoxic T lymphocytes (CTLs) and CD4+ helper T cells. Kumagai et al. reported that PD-1 inhibitor treatment benefited patients with lung and gastric cancers and high and low PD-1 expression on effector T cells and Tregs, respectively (33).

Extracellular acidosis suppresses T cell-mediated immunity, and neutralization of tumor acidity reportedly improves antitumor responses to immunotherapy. Lowering the pH of the TME likely induces anergy in human and mouse tumor-specific CD8+ T cells through mTORC1 inhibition, thereby reducing cytolytic activity and cytokine production (34).

Several studies have reported on the effect of lactate on T cells, which is the primary cause of TME pH reduction. Many effector T cells are inactivated by glucose depletion and elevated lactate levels triggered by tumor cells, as their proliferation and cytokine production are highly dependent on glycolysis (35). Inhibition of glycolysis in CD4+ helper T cells and CTLs also reduces cell motility associated with decreased responsiveness to chemokines (36). Furthermore, the high lactate concentration in the TME inhibits lactate efflux from T cells, thereby reducing cytokine production and cytotoxic activity (37, 38). In contrast, in Treg cells, the master transcription factor forkhead box P3 (FOXP3) makes energy production less reliant on glycolysis and more on oxidative phosphorylation, which improves survival and maintains immune suppressive function in low-glucose and high-lactate environments (39, 40). Thus, TME lactate elevation reduces effector T cell function and attenuates anti-tumor immunity without affecting Treg cell function. Furthermore, lactate in the TME reduces the release of soluble granule contents such as perforin and granzyme from NK cells, decreases the production of cytokines such as IFN-γ and TNF-αand indirectly suppresses NK cell function by increasing MDSCs (41–43). Moreover, the effects of the acidic TME on NK cells are reversible: oral administration of bicarbonate to a lymphoma mouse model and raising the TME pH to the physiological pH of 7.2–7.5 increased the production of IFN-γ by NK cells and suppressed tumor growth (44).

## Adaptation of macrophages to the acidic tumor microenvironment

Macrophages are divided into M1 and M2 phenotypes. M1 macrophages are responsible for innate immune responses through the secretion of inflammatory cytokines, phagocytosis of foreign substances, and the presentation of antigens. They are involved in Th1-type responses. Th1 cytokines such as IFNγ and IL-12 and foreign antigens such as lipopolysaccharide (LPS) induce differentiation to the M1 phenotype. Conversely, M2 macrophages are induced by Th2 cytokines such as IL-4, IL-10, and IL-13 and play pivotal roles in immunosuppression, tissue remodeling, and angiogenesis. TAMs often exhibit M2-like traits in many malignant tumors and act as tumor promoters (45). IL-10 and TGF-β secreted by TAMs suppress Th1, as well as induce regulatory T cells, thereby suppressing T cell immune responses (46).

An acidic TME favors polarization to M2 macrophages *in vitro* and *in vivo* and additionally increases angiogenic vascular endothelial growth factor (VEGF) production (47, 48). The lactate-induced M2 macrophage polarization reportedly involves the ERK-STAT3 signaling pathway (49), HIF1α stabilization (50), and G protein-coupled receptor 132 (GPR132) activation. Furthermore, Zhang et al. reported that post-translational modification of histone proteins by lactyl groups derived from lactate induces M2 polarization (51). Furthermore, lactate inhibits monocyte differentiation into dendritic cells, and high lactate levels in the TME may interfere with dendritic cell formation and accumulation (52).

## Adaptation of myeloid-derived suppressor cells to the acidic tumor microenvironment

Myeloid-derived suppressor cells (MDSCs) are classified into granulocytic/polymorphonuclear MDSCs (PMN-MDSCs) and monocytic MDSCs (M-MDSCs) according to their origin. A hallmark of MDSCs is their ability to inhibit immune responses, including those mediated by T, B, and NK cells. M-MDSCs and PMN-MDSCs share features that facilitate suppression of immune responses, including the activation of STAT3 expression, induction of ER stress, expression of arginase 1, and expression of S100A8/A9. Furthermore, PMN-MDSCs preferentially use reactive oxygen species (ROS), peroxynitrite, arginase 1, and prostaglandin E2 (PGE2) to mediate immunosuppression, whereas M-MDSCs mediate nitric oxide (NO), induce immunosuppression through immunosuppressive cytokines such as IL-10 and TGFβ and immunomodulatory molecules such as PDL1 (53).

MDSCs reportedly upregulate PD-L1 expression and PD-1-mediated suppression of T cells through the lactate-induced HIF1α pathway in TMEs (54). Furthermore, MDSCs may promote the formation of tumor blood vessels by enhancing the production of angiogenic factors such as VEGF through the lactate-induced HIF1α pathway in TMEs, further contributing to the hypoxic conditions (43). VISTA, an immune checkpoint molecule expressed in MDSCs, is directly induced by acidification, resulting in immunosuppression (55).

## Adaptation of cancer-associated fibroblasts to the acidic tumor microenvironment

In some cancers, such as breast and pancreatic cancers, cancer-associated fibroblasts (CAFs) are the most prominent stromal cell type, and their presence is associated with a poor prognosis. They have various origins, including resident tissue fibroblasts educated by primary cells, mesenchymal cells recruited from the bone marrow to the TME, and adipocyte-derived progenitor cells. The functions of CAFs in the TME are also diverse and participate in promoting tumor progression, including direct cancer cell proliferation, immunosuppression, angiogenesis, and promotion of extracellular matrix (ECM) remodeling. These functions are mediated by complex reciprocal signaling interactions with cancer cells, the ECM, and infiltrating immune cells (56).

CAF directly interacts with prostate cancer cells to promote lactate production through the expression of the glucose transporter GLUT1 and to induce TME acidification by releasing lactate *via* monocarboxylic acid transporter-4 (MCT4). Simultaneously, it induces Th1 cell suppression and Treg-induced immunosuppression (57, 58). Thus, CAF promotes metabolic-based tumor growth with TME acidification by interacting with tumor cells.

## Approaches of alkalization of the acidic TME

As described above, TME acidification by cancer cells is considered to be one of the immune escape mechanisms and causes poor clinical outcomes. Therefore, in addition to alkalizing agents such as bicarbonate, inhibitors against membrane-bound proton transporters, such as NHE1, Na+/K+ ATPase pump, V-ATPase, H+/Cl− symporter, MCT, and CA have been attempted to be developed as alkalizing therapy for TME (59, 60). In clinical practice, there is a report that the prognosis was improved by alkalizing therapy, using urinary pH as an indicator of alkalinization (61–63). In addition, CAIX inhibitors, which are intensively researched (64), have been reported to enhance ICI antitumor effects in preclinical models, and clinical applications of combined immunotherapy and alkalizing therapy are expected in the future.

## Conclusion

Tumors exploit the local acidification using lactate to interact with the cells that constitute the TME and facilitate immune escape, which involves the suppression of immune cells with anti-tumor activity, activation of immunosuppressive cells, and promotion of the malignant transformation of CAF-forming stroma and proliferation of tumor blood vessels. The development of therapeutics that inhibit pH-responsive proteins, such as MCT, and the administration of buffers to adjust the pH level of the TME may be further explored as potential therapeutic alternatives.

## Author contributions

MH and KY wrote the manuscript. Both authors contributed to the article and approved the submitted version.
